# Which Environmental Pollutants Are Toxic to Our Ears?—Evidence of the Ototoxicity of Common Substances

**DOI:** 10.3390/toxics12090650

**Published:** 2024-09-04

**Authors:** Gregory M. Zarus, Patricia Ruiz, Rae Benedict, Stephan Brenner, Krystin Carlson, Layna Jeong, Thais C. Morata

**Affiliations:** 1 Agency for Toxic Substances and Disease Registry, Office of Innovation and Analytics, Atlanta, GA 30341, USA; pruiz@cdc.gov (P.R.); rbenedict@cdc.gov (R.B.); sbrenner@cdc.gov (S.B.); 2 National Institute for Occupational Safety and Health, Cincinnati, OH 45226, USA; kcarlson@cdc.gov (K.C.); tmorata@cdc.gov (T.C.M.); 3 Georgia Tech School of Biological Sciences, Atlanta, GA 30332, USA; hjeong308@gatech.edu

**Keywords:** ototoxicity, hearing loss, environmental pollutants, toxicological profiles, evidence review

## Abstract

Ototoxicity refers to the adverse effects of substances on auditory or vestibular functions. This study examines the evidence of ototoxicity’s association with exposure to common environmental pollutants, as documented in toxicological profiles by the Agency for Toxic Substances and Disease Registry. Our aim was to evaluate whether the evidence supports modifying the charting of ototoxic effects in the summary tables of these toxicological profiles and providing a guide for scientists to access these data. Health outcomes of interest included hearing loss, vestibular effects, cochlear lesions, tonal alterations, cellular damage, and ototoxicity-related outcomes (neurological, nephrotoxic, hepatic, and developmental effects). We obtained ototoxicity information for 62 substances. Hearing-related effects were reported, along with neurological effects. Overall, 26 profiles reported strong evidence of ototoxicity, including 13 substances previously designated as ototoxic by other health and safety agencies. Commonly studied outcomes included hearing loss, damage to ear anatomy, and auditory dysfunction. Vestibular dysfunction and tinnitus are rarely studied. Our findings highlight the lack of conclusive evidence of ototoxic properties for many substances, especially for pesticides and herbicides. This review supports charting the evidence of ototoxicity separately in toxicological profiles’ summary tables. Improving the communication of ototoxicity-related health effects might impact their recognition and prompt further research. A stronger evidence base could support improved prevention efforts in terms of serious health outcomes.

## 1. Introduction

Many toxic substances used in workplaces can lead to detrimental neurological effects in humans. Some of these substances are known to be ototoxic and can impact the human sensory systems of the inner ear and lead to vestibular dysfunction or hearing loss. While research on these substances’ ototoxicity is largely confined to workplace settings, most of these chemicals are also found as pollutants in the environment. Thus, their neurotoxic and ototoxic properties could potentially affect the population at large. In dealing with the body of literature on substances and their association with hearing impairment, there is need for a deeper review and summary of ototoxic chemicals considering the existing evidence. We examined the evidence of ototoxicity associated with exposure to common environmental pollutants in the toxicological profiles of the Agency for Toxic Substances and Disease Registry. We aimed to evaluate if the evidence supported a modification of the charting of ototoxic effects in the toxicological profiles’ summary tables.

### 1.1. Ototoxicity and Health Burden

Ototoxicity (i.e., impairment or loss of hearing and/or balance due to exposure to chemical or physical pollutants) represents an important public health concern. Hearing loss alone has become the most reported sensory impairment among Americans [1,2,3]. In the U.S., 13.0% of adults have hearing loss, 9.6% experience tinnitus, and 35.4% over the age of 40 suffer from simple postural metric vestibular dysfunction [4,5,6]. Exposure to ototoxic substances can affect different functions of the inner ear (e.g., sense of unsteadiness, dizziness, and tonal changes). The diagnosis, treatment, and rehabilitation of ototoxic effects typically requires a complex team of specialists, including audiologists, otolaryngologists, hearing aid specialists, neurologists, and physical therapists [7].

The total health burden related to ototoxicity is not yet entirely understood, as secondary health effects cause or contribute to the development of other health outcomes (e.g., cognitive impairment, psychosocial health problems) throughout life. For instance, children with hearing loss are at risk for delayed language and psychosocial developments if the condition is not detected and managed early [8,9]. Similarly, children with vestibular dysfunction have an increased risk for delayed motor development [10]. Adults suffering from tinnitus or other hearing deficits often exhibit lower workplace performance and higher levels of anxiety, depression, and even suicide [11]. Older adults with untreated hearing loss often lose other cognitive abilities as well and have a higher risk for developing dementia [12]. Given these delayed secondary effects, the overall health burden experienced due to hearing loss and vestibular dysfunctions is challenging to assess.

### 1.2. Exposure to Ototoxic Substances

Ototoxic exposure contributes significantly to the development of hearing loss. However, newer substances, introduced annually, have often not undergone any neurotoxic testing. Even for substances that are tested for their neurotoxic potential, effects related to hearing impairment, dizziness, or imbalance are often classified and reported under the generalized term of “neurotoxic effects”, which might mask existing evidence on the specific ototoxic properties of some of these substances.

**Noise**, as a physical ototoxicant, has been well studied and is believed to be the greatest contributor to acquired hearing loss. About 12.5% of children and adolescents aged 6–19 years, as well as 17% of adults aged 20–69 years, are estimated to have permanent damage due to excessive noise exposure [13,14]. Environmental noise might also intensify the negative effects of other toxic chemicals. For instance, a recent study in children suggested that environmental exposure to benzene in combination with environmental noise increased hearing loss [15]. Similarly, the combination with noise increased the nephrotoxic effects of toluene in animal studies [16].

Several **pharmacological drugs**, such as diuretics, aminoglycoside antibiotics, anti-inflammatory agents, and antineoplastic agents, are associated with tinnitus and acute and transient hearing impairment [17]. Platinum-based antineoplastic drugs, such as cisplatin or carboplatin, have been studied well. Cisplatin accounted for hearing loss in half or more of the patients treated with this drug, alone or in combination with carboplatin, which translates to about half a million people with drug-induced hearing loss [18].

**Occupational exposure** to ototoxicants is common in industries related to manufacturing, mining, utilities, construction, and agriculture. Manufactured products that involve or contain ototoxic chemicals include paints, plastics, petroleum, solvents, fabricated metals, machinery, leather, textiles, paper, furniture, ships, electrical equipment, appliances, solar cells, and batteries [19]. Exposure to non-pharmaceutical ototoxicants usually results from workplace exposure, most typically in industrial settings. Occupational exposure to styrene, for instance, has been linked to hearing loss, with the ototoxic effect potentiated in the presence of noise [20]. Occupational exposure to solvents or jet fuels has been linked to vestibulo-oculomotor and auditory abnormalities [21,22]. Other ototoxicants, such as ethyl benzene, toluene, styrene, xylene, trichloroethylene, lead, and mercury, are among substances frequently found in the environment [23,24].

### 1.3. Disorders Associated with Ototoxic Substance Exposure and Hearing Impairment

Most ototoxic substances that affect the auditory system have **additional organotoxic effects**. For instance, ototoxic substances such as polychlorinated biphenyls (PCBs) have been found to not only impact hearing functions in the inner ear, but also to affect auditory processing within the central nervous system, a feature shared with other known neurological disorders [25,26]. Some ototoxic substances in our environment, such as lead, mercury, tin, zinc, dioxins, PCBs, and polycyclic aromatic hydrocarbons (PAHs), represent known neurotoxicants. Some authors suggest that each of these substances, which have neurotoxic mechanisms, may be involved in the pathogenesis of neurodegenerative disorders like Alzheimer’s disease, Parkinson’s disease, or amyotrophic lateral sclerosis (ALS) [27,28,29,30,31]. Similarly, a few ototoxicants, such as toluene and some heavy metals, have known nephrotoxic or carcinogenic effects [16,32,33,34].

Exposure to some ototoxic substances, such as PCBs, lead, or cadmium, before birth or during infancy can also result in **developmental defects** in the auditory system [25,35]. Initially formed as cartilage at six weeks of gestation, the middle ear bones of humans continue to develop and ossify throughout pregnancy [36]. PCBs, several metals, and pesticides have been found to be toxic to bone development during gestational days 9–13 in mice, which translates to gestational weeks 4–9 in humans [37]. The differentiation of hair cells in the cochlea begins around 10–12 weeks of gestation, the period during which several pesticides, metals, and some volatile organic compounds (VOCs) are known to negatively interfere with the neurological development of a fetus [37,38].

Associations may also exist between environmental substance exposure and **genetic factors** (e.g., catechol-O-methyltransferase (COMT) polymorphism) in the development of hearing loss, neurobehavioral disorders, and depression [39,40,41,42,43]. This suggests the impact of environmental substance exposure on genes linked to hearing loss or other neurological effects. While the exact role of environmental substances and the mechanisms by which they contribute to the development of these neurological disorders are unknown, environmental neurotoxicants and genotoxic pathways seem to share common endpoints.

Several **organ-specific disorders** and syndromes involve the auditory system. For instance, progressive familial intrahepatic cholestasis (Byler disease), fatty liver disease, and viral hepatitis are linked to sensi-neuronal hearing loss [44,45,46]. Sensi-neuronal hearing loss is also common among patients with advanced stages of kidney disease and reduced renal function [47,48,49]. Further, several hereditary kidney abnormalities, such as Alport syndrome, are characterized by both ear and kidney abnormalities [50,51].

## 2. Materials and Methods

**Figure 1:**
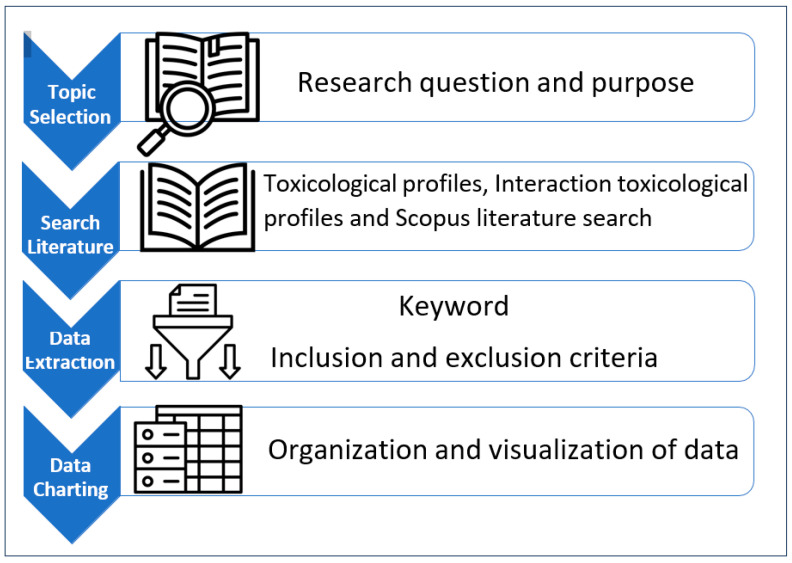
Sequence of methodological steps of evidence review.

Figure 1 provides a graphical summary of the methodological steps taken by the authors. This study was based on a review of toxicological or interaction profiles (federal documents), which summarize toxicological evidence on selected environmental substances and are published by the U.S. Agency for Toxic Substances and Disease Registry (ATSDR). These profiles served as the main source for this review, as they contain direct and indirect evidence of ototoxic properties for each profiled substance. These profiles were also checked for the recency of ototoxic information. In addition to ototoxic information, each profile was further reviewed for information on liver, kidney, neurological, and developmental outcomes to assess evidence on whether ototoxic substances cause these other related health outcomes.

### 2.1. ATSDR Toxicological Profiles

As part of its mission to protect communities from harmful health effects related to exposure to natural and man-made hazardous substances, ATSDR has been mandated to prepare toxicological profiles on those hazardous substances most frequently found at waste sites [52]. Each profile provides a comprehensive summary of available evidence on the known health effects of each substance [53]. Toxicological profiles are developed for substances based on their ranking in ATSDR’s Substance Priority List (SPL) [54]. This priority list is developed in close coordination with the Environmental Protection Agency’s (EPA’s) National Priorities List (NPL) of important hazardous waste sites requiring remediation [55]. The SPL therefore includes substances found at these sites and prioritizes them according to their toxicity, occurrence, and completed human exposure pathways. In addition, ATSDR publicly announces and accepts nominations from individuals, organizations, or agencies for consideration in toxicological profile development.

Each toxicological profile periodically undergoes a review to ascertain whether there is any new scientific evidence which could spur its update. In addition to the title substance, a profile may review and address isomers and compounds associated with the title substance, as some isomers or compounds might have toxic profiles that differ substantially or have been studied more thoroughly compared to the title substance. For instance, the xylene profile addresses three different xylene isomers (ortho, meta, and para), and the lead profile addresses a range of lead species and compounds (e.g., lead chromates, lead oxides, lead sulfates, lead acetate, tetraethyl lead). ATSDR also develops chemical interaction profiles for substances that are commonly found in mixtures. 

In preparing or updating profiles, ATSDR systematically gathers and reviews all published research available on the health effects associated with the substances. The reviews include gray literature which ATSDR has peer-reviewed, as explained in Appendix B of each profile. Each profile compiles information on the substance’s potential health effects, physicochemical properties, use, potential for human exposure, toxicokinetics, biomarkers, and minimal risk levels (MRLs). Substance-specific MRLs are defined as “an estimate of the daily human exposure to a hazardous substance that is likely to be without appreciable risk of adverse noncancer health effects over a specified duration of exposure” [56]. MRLs provide screening levels to allow public health professionals to identify contaminants and potential effects after exposure to a given substance [57]. Profile drafts undergo several reviews by CDC/ATSDR experts, interagency (e.g., EPA) reviewers, and non-government independent peer reviewers. Following revisions, profiles are announced in the Federal Register and posted on the web for 90 days, during which time the public may submit comments. Finalized profiles are posted online to be accessed and used by the public for free (http://www.atsdr.cdc.gov/toxprofiles/index.html (accessed on 28 August 2024)).

### 2.2. Toxicological Profile Selection Criteria

These literature searches were conducted from June 2022 to June 2024. This time corresponds to a particular ranking of substances on the SPL [55]. At the time of this review, a total of 184 toxicological profiles and 16 interaction profiles had been published. Profile selection was guided by the following inclusion and exclusion criteria: 

We included 53 profiles of substances belonging to chemical classes or being involved in chemical interactions with high ototoxic potential based on occupational studies, high SPL rankings, and a reasonably recent publication date. This included substances commonly classified as VOCs/solvents, pesticides/herbicides, and metals. These 53 profiles included 15 profiles released as final editions or drafts for public comment in 2022 or later.

We included all five profiles on fuel and oils given the known ototoxic potential of many petroleum-based products. We included all four profiles of small-molecule sulfur substances, as these represent common air pollutants. We conducted substance screening using the same terms in Scopus (https://elsevier.libguides.com/Scopus/topical-search (accessed on 28 August 2024)) as described above to ensure the profiles included much of the relevant data. We considered excluding those substances for which there were many recent studies on ototoxicity that had not been vetted by ATSDR’s process. Some substances were not excluded through this process as many of the recent citations were found in other substance profiles, as was the case with fuels and some pesticides.

While some candidate substance groups were excluded from charting, we provided references to them in our discussion because of their ototoxic potential. Most notably, we excluded PCBs, which include a total of 209 chemicals, of which many have ototoxic potential. Released in 2000, the profile on PCBs includes ototoxic evidence on a few PCB substances. However, additional research on PBCs has been conducted since then that has not yet been systematically reviewed as part of ATSDR’s profile development. Given the large number of PCBs, their ubiquitous presence in our environment, and presence in many commercial and consumer products, as well as their vast chemical variability, PCBs deserve a separate review with other similar persistent organic pollutants.

For this review, we identified a total of 60 toxicological profiles and 1 interaction profile for *Benzene, Toluene, Ethylbenzene, and Xylene (BTEX)* [58]. Although the *Toxicological Profile for Hydrogen Sulfide and Carbonyl Sulfide* is a single publication, we treated its subjects as two separate substances in our analysis given their distinct toxicological differences. This resulted in a total of 62 distinct substances or substance groups being considered for review. We did not separate the other combined substance profiles, such as those for substances categorized as pesticides and herbicides (i.e., DDT, DDD, and DDE; aldrin and dieldrin, heptachlor and heptachlor epoxide; or phosphate ester flame retardants), further given the lack unique substance-specific findings. We classified these 62 substances or substance groups into five broader categories and provided citations of the profiles within each category alphabetically: fuels and oils [59,60,61,62]; volatile organic compounds (VOCs) and solvents [63,64,65,66,67,68,69,70,71,72,73,74,75,76,77,78,79,80,81]; pesticides, herbicides, and chemical pest barriers [82,83,84,85,86,87,88,89,90,91,92,93,94,95,96,97,98,99,100,101,102]; sulfides [103,104,105]; and metals [106,107,108,109,110,111,112,113,114,115,116,117,118].

### 2.3. Data Extraction and Charting

Charting consisted of a detailed reading of each profile to identify direct or indirect evidence indicating the research findings on ototoxicity and related organ system toxicity. The charting of a profile for an effect was based on human or laboratory mammal research with respect to six health effect categories: hearing impairment, vestibular effects, tinnitus, inner ear effects, other major organ systems, and early developmental effects. We first reviewed each substance profile for any reported information on ototoxicity and/or ototoxicity-associated health effects, using the following key words or phrases for each health effect category:

Hearing impairments: “hearing loss”, “speech frequency loss”, “other frequency hearing loss”, and “tonal alteration”.

Vestibular effects: “dizziness”, “unsteadiness”, “spatial disorientation”, “vertigo”, “oscillopsia”, “nystagmus”, “saccades”, “involuntary eye motion”, “other visual system disharmony”, “headache”, “equilibrium”, “balance”, “incoordination”, and “ataxia”.

Tinnitus: “ringing”, “buzzing”, “throbbing”, and “other repeating tones”.

Inner ear tissue and cells: “cochlear hair cells”, “cochlear supporting cells”, “utricular hair cells”, and “utricular supporting cells”.

Other major organ systems associated with hearing or balance loss: “liver”, “hepatic system”, “hepatotoxicity”, “kidney”, “renal system”, “nephrotoxicity”, “nerve”, “nervous system”, and “neurotoxicity”.

Developmental effects (e.g., otitis media) related to hearing loss: “developmental effects”.

The identified content for health effects, exposure routes, and evidence levels was extracted and charted using an Excel datasheet, which allowed us to organize, compare, and summarize relevant findings across profiles and substances. The authors then evaluated and extracted information based on the level of evidence provided in the underlying information source (e.g., the specificity of effects such as damage to cells, dose–response relationships, and the direct measurement of auditory loss). We used heat mapping to visually indicate both the specificity of the evidence and the relevance or severity of the assessed health outcome. Summaries of the unique suggestive evidence are provided in the Results and Discussion sections.

In a next step, we reviewed the substance designations provided by seven different health organizations to verify our substance selection and to identify any additional substances using existing evidence on ototoxicity. This additional review included the listed information provided by the following organizations or institutions:

American Counsel Government Industrial Hygienists (ACGIH).

American Speech-Language-Hearing Association (ASHA): “Chemicals That Affect Hearing & Balance” [119].

Canadian Centre for Occupational Health and Safety (CCOHS): “Occupational Hygiene—Ototoxic Chemicals” [120].

European Agency for Safety and Health at Work (EU-OSHA): “Combined Exposure to Noise and Ototoxic Substances” [121].

UK Hearing Conservation Association (HCA): “Ototoxicants—what are they and how may they worsen hearing loss in the workplace?” [122].

U.S. Occupational Safety and Health Administration (OSHA)/U.S. National Institute for Occupational Safety and Health (NIOSH): “Preventing Hearing Loss Caused by Chemical (Ototoxicity) and Noise Exposure” [123].

In our extraction sheet, we assigned an additional label to those substances identified from the profiles in the above external sources as designated ototoxicants. Any additional information on these substances on auditory or non-auditory health effects and related evidence levels was extracted and compared to the information extracted during the profile review. We then labeled substances as having evidence of ototoxicity (EO) or as having some evidence of ototoxic potential (SEOP) based on the strength of the evidence identified. Because studies on tinnitus and vestibular function were rare, EO substances largely represented substances for which evidence of hearing loss was identified by studies designed to specifically measure this health outcome. Damage to cochlear cells was often the strongest evidence. Specific cases of SEOP are addressed in the discussion section.

## 3. Results

As described above, we reviewed a total of 62 profiles for evidence of ototoxic and ototoxic-related health effects. The profiles contained distinct substances or substance groups included in the SPL. Table 1 summarizes ototoxic and related associations between each substance or substance group, organized into five groups: fuels and oils (5 profiles), volatile organic compounds (VOCs) and solvents (19 profiles), sulfides (4 profiles), pesticides, herbicides, and barriers (21 profiles), and metals (13 profiles). Our heat mapping included four color tones (red, yellow, blue, gray) to suggest the degree of evidence for the ototoxic effects. Red fill indicates strong evidence (EO) and yellow indicates possible or suggestive evidence (SEOP). The cooler colors, blue and gray, indicate little or no evidence and negative findings, respectively. We use a bold font to identify substances that have a confirmed hearing ototoxicant designation by one or more of the health and safety organizations reviewed (first and last columns). In addition to these agencies’ findings, when the scoping review identified new important ototoxicity findings, they were indicated in the last column, entitled “Ototoxicity Confirmation and Supporting Data”.

The substances are linked to their online profiles in the Toxic Substances Portal. This portal will add newer profiles as more research on health effects becomes available.

* = the current profile does not include hearing loss data, but the chemical is identified with hearing loss evidence in another profile (or other cited reference).

**Evidence ranking**: Y = yes; P = possible; NA = no data available; NS = studies are not specific; Neg = negative findings; DL/IF = data lacking or inconsistent findings; equilib = equilibrium effects.

**Exposure routes**: I = inhalation; O = oral; D = dermal.

**Color coding and degree of ototoxic evidence**: red—strong evidence; yellow—possible or suggestive evidence; blue—little or no evidence; gray—negative findings.

**Bolded substances** are recognized by one or more of the following organizations as an ototoxicant.

HCA = UK Hearing Conservation Association 

OHS = Occupational Health and Safety

OSHA = Occupational Safety and Health Administration

ASHA = American Speech-Language-Hearing Association

NIOSH = National Institute for Occupational Safety and Health

CCOHS = Canadian Centre for Occupational Health and Safety

### 3.1. Levels of Reported Ototoxicity Evidence

Of these profile-specific substances, 13 (about 20%) were designated as ototoxicants (OTOs) by the reviewed health and safety agencies. These included eight VOCs, one pesticide (cyanide), one sulfide (carbon disulfide), and three metals. None of the substances listed by us under fuels and oils were designated as ototoxicants by the external sources reviewed. 

We identified 26 profiles which reported strong EO. These included all 13 designated ototoxicants mentioned above. We further identified 41 profiles which reported SEOP and the SEOP category included all 26 EO profiles. These two designations are separated by shading in the summary table, with details provided in the profiles—often within Section 2 and Section 3—and at times explained in detail in other sections. Evidence was considered suggestive when clear evidence on the mechanisms or study specificity was limited. Examples of the SEOP category included the following metals and issues: cobalt, which shows limited evidence of a decrease in auditory response during cobalt therapy [110]; chromium, with suggestive evidence of auditory damage in rats [128]; chloroform-induced inner ear damage in hamsters [124]; and selenium, with high levels found to be associated with hearing loss [132].

### 3.2. Evidence for Ototoxic Health Effects

Substance-induced ototoxicity was reported to have effects on several sites within the hearing structures by way of measurement. The studies reported the function of the inner ear’s auditory and vestibular apparatus and the associated neural pathways. Evidence of associations between the reviewed substances and hearing dysfunction was limited overall, and even more so for vestibular effects or tinnitus. As shown in Table 1, we identified 26 substance profiles with strong evidence of hearing loss, including 2 profiles grouped under fuels and oils, 12 under VOCs, 5 substances under pesticides and herbicides, 1 under sulfide, and 7 under metals.

We identified eight substance profiles with evidence of vestibular effects, including one grouped under fuels and oils, five under VOCs, and two under metals. We found no evidence for tinnitus in any of the reviewed profiles.

Of the 15 profiles published between 2022 and 2024, only 7 (47%) included evidence of ototoxicity and 2 others (i.e., the 1,1,1-Trichloroethane Profile and Aldrin and Dieldrin Profile) included studies reporting no ototoxicity (noted with neg. in Table 1). Of note, none of the reviewed profiles provided an MRL that was specifically developed for hearing loss, vestibular effects, or tinnitus.

**Fuels and oils**: Of the five profiles categorized under fuels or oils, a strong EO was reported in two profiles (i.e., Jet fuels A, JP5 and JP8 Profile and BTEX Interaction Profile), and SEOP was found for another two (i.e., Jet Fuels JP4 and JP7 Profile and Fuel Oil and Kerosene Profile). None of the substances included in this category were designated as OTOs.

**VOCs and solvents**: Of the 19 substance profiles categorized under VOCs and solvents, strong EO was reported by 11 profiles, among which 8 substances (benzene, toluene, ethylbenzene, xylenes, chlorobenzene, styrene, trichloroethylene, and n-hexane) were designated as OTOs. These substances are commonly found in fuels, paints, and solvents. Five other VOCs and solvents had SEOP. Three substance profiles reported no research on ototoxicity (Acrolein Profile, 2-Butanone Profile, and 1,2-Dichloroethane Profile). However, both the 2-Butanone and the 1,2-Dichlorethane profiles reported evidence of neurotoxicity.

**Pesticides, herbicides, and barriers**: Most of the substances and substance groups categorized as herbicides, pesticides, and barriers have not been examined for their ototoxic properties. Only five profiles (about 24%) reported SEOP, but only one substance, cyanide, has been listed as a designated OTO by an occupational health organization.

**Sulfides**: While substances in this category could have also been categorized as solvents or pesticides, we considered them to be a separate category in this review, as they occur naturally in the environment due to decay, degradation, or combustion and thus are commonly associated with landfill gases. For the four sulfides addressed here (carbonyl sulfide, carbon disulfide, hydrogen sulfide, and sulfur dioxide), reported evidence has been overall limited and disparate with respect to ototoxic evidence. Strong EO was only reported for carbon disulfide.

**Metals**: Almost all reviewed metal profiles (12 out of 13) reported some level of ototoxic evidence; nickel was the exception. More than half (7 out of 13) of the profiles reported robust studies associated with hearing loss, with three of these substances designated as OTOs by an occupational health organization. Many of these metals are part of chemical compounds found naturally in the soils or are used in several industries.

### 3.3. Potential Relationships between Ototoxicity and Other Major Health Effects

Of the 62 substance profiles reviewed, 42 (nearly 70%) reported neurological effects other than auditory or vestibular. Substances designated as OTOs by all (100%) organizations were confirmed to be neurotoxicants by their respective ATSDR profiles. Table 2 provides a summary of the relationships between ototoxic and non-ototoxic effects below.

**Neurotoxicity**: All 13 organization-designated OTO substances showed evidence for neurological effects, including neuromotor, cognitive, and sensory effects. Overall, 89% (25/26) of the EO substances presented other neurological effects, and 85% (35/41) of the substances with SEOP also presented other neurological risks.

**Renal and Nephrotoxicity**: About half (7 out of 13) of the OTO substances reported evidence of renal and nephrotoxic effects. Among the 26 substances with strong evidence of ototoxic effects, 13 profiles also reported nephrotoxic properties. Among the 41 SEOP substances, 17 profiles reported nephrotoxic effects.

**Hepatotoxicity**: About 46% (6 out of 13) of the OTO substances reported evidence of hepatic effects. Among the 27 substances with strong EO, 13 profiles (44%) also reported hepatotoxic effects. Among the 41 SEOP substances, 20 profiles (49%) reported liver effects.

**Developmental effects**: Overall, 5 out of the 13 OTO substances (38%) reported evidence of developmental effects. Among the 26 substances with strong EO, 10 profiles (37%) were also reported to affect auditory development. Among the 41 SEOP substances, 12 profiles (29%) also reported developmental risks.

**OTO 13** represents the 13 (of 62) substances designated as ototoxic by other environmental health and safety organizations.

**EO 26** represents the 26 (of 62) substances identified as having evidence of ototoxicity in their profiles, including the OTO 13.

**SEOP 41** represents the 41 (of 62) substances with evidence suggestive of ototoxic potential, including the EO 26.

**Header color coding and association with ototoxicity:** red—strong association; yellow—moderate association; green—lower association; blue—lowest association. 

## 4. Discussion

We reviewed and summarized the current evidence of the ototoxic effects of hazardous substances with high risks of environmental and occupational exposure. We further highlighted the evidence of the association of these substances with health effects related to general neurotoxicity and four non-auditory organ systems commonly involved in patients diagnosed with hearing loss. Our findings suggest that studies on the ototoxic effects of environmental or occupational chemicals are commonly restricted to health outcomes related to hearing loss. In contrast, outcomes such as tinnitus and vestibular dysfunctions are less often investigated and less commonly reported among neurotoxic effects more generally [26,133].

In the substances reviewed here, ototoxicity-related health outcomes were generally less studied than other health outcomes. Few profiles identified more than four studies on hearing loss, especially regarding pesticides, while most profiles did not report any ototoxicity-specific studies. Yet, no dosing studies have been performed for individual substances to determine safe levels with respect to ototoxic effects. Furthermore, vestibular-specific dizziness is rarely investigated, and our review identified only one study associating workplace exposure to lead, toluene, and carbon monoxide with tinnitus [134].

This stands in stark contrast to the hundreds of studies reviewed and cited on hepatic, developmental, neurological, renal, and other effects for most of these substances. It is important to note that ototoxic substances commonly pose other neurotoxic risks, including cognitive, neuromotor, and other neurosensory effects, and cause other significant and serious health effects. While details on the dose–response relationships and substance-specific mechanisms for non-ototoxic outcomes are frequently reported by their respective substance profiles, this is not yet common practice for ototoxic effects and this is likely a result of the limited number of ototoxicity studies, especially for pesticides, herbicides, and chemical barriers.

While our findings on substance-specific ototoxicity did not necessarily identify new causal links between substance exposure and health endpoints, they nevertheless highlighted the vast evidence gap in ototoxicity-specific research for most, if not all, potential ototoxicants commonly found in our environment. Our findings further illustrate three different relationships:

First, the relationship between substance exposure and hearing loss.

Second, the relationship between hearing loss and other ototoxic and non-ototoxic effects.

Third, the relationship between exposure to ototoxic substances and non-ototoxic health effects.

No profiles have identified dose–response links addressing all three of these relationships. This is a major data gap in terms of developing safe exposure and medical monitoring guidance.

### 4.1. Ototoxicity by Substance Category

#### 4.1.1. Fuels and Oils

Based on blood samples obtained from the general population, most persons show signs of fuel exposures [135,136,137]. Occupations involving hydrocarbon fuels have a known higher risk of hearing loss [22,24,138,139]. Recent studies on benzene biomarkers suggest that even children are environmentally exposed to fuel levels that can result in hearing loss [15]. While it is difficult to isolate substance-specific exposures when assessing occupational exposures to fuel in worker studies, existing animal-based research is more suggestive. In case control studies with animals, comparing exposure to kerosene-based fuels vs. exposure to synthetic fuel without aromatic hydrocarbons, the kerosene fuels resulted in a clear ototoxic effect and the synthetic fuel showed little or no ototoxic effect [140,141,142].

#### 4.1.2. VOCs and Solvents

Industrial workers are exposed to numerous solvents across many industries. Many of these substances are also frequently found at low levels in the ambient air—especially near industrial centers and landfills—and found in the groundwater of hazardous waste sites [143]. Some communities near contaminated waste sites are exposed to vapor, moving from contaminated groundwater into their homes. In one study, 15 VOCs were present above known safety levels [144], including 7 known or suspected ototoxic substances: trichloroethylene (TCE), tetrachloroethylene (PERC), chloroform, benzene, toluene, ethylbenzene, and xylene. Studies on TCE, PERC, chloroform, toluene, ethylbenzene, and xylene showed evidence of specific cochlear cell effects associated with hearing loss [124,145,146,147,148,149,150,151,152,153,154,155,156]. While the mechanism of ototoxicity is less clear, exposure to benzene is associated with both vestibular dysfunction and hearing loss [15,157,158,159,160]. Although no substances have sufficient data to develop an MRL based on hearing loss, vestibular function, or tinnitus, research on two VOCs provides sufficient data to develop an MRL based on some search term word effects. The acute inhalation MRL, defined in the 2-Butanone Profile, involves neurological symptoms that include headache, and the tetrachloroethylene (PERC) profile’s chronic inhalation MRL relates to color vision.

#### 4.1.3. Pesticides and Herbicides

The scarcity of dose–response studies for single pesticides is remarkable. Occupational studies have identified pesticide workers as having elevated risks for hearing loss, tinnitus, and other ototoxic effects [161,162,163,164,165]. With few animal studies on pesticides, their mechanisms of ototoxicity have not yet been fully assessed. However, organochlorine and organophosphate pesticides are known to induce oxidative stress, DNA damage, and inflammatory responses [161,162,163,164,165].

Further, organophosphate, chlorinated, and pyrethroid insecticides used to combat insects and their larvae have been associated with a higher risk of hearing loss (peripheral and central) and balance disorders [161,166,167,168,169]. Most of these substances are only available for licensed contractors to purchase, yet have been applied to residential and commercial crops. Treated wood products contain pentachlorophenol (PCP) or other pest barriers. Older treated wood homes included PCP [170], and communities near treatment facilities can be exposed to PCP in the air [171,172,173,174]. Despite these facts, the potential risk seems to remain unrecognized and medical surveillance of auditory outcomes has yet to be mentioned by occupational health agencies.

#### 4.1.4. Sulfides

While the sulfides reviewed could also be classified as solvents or pesticides, these substances represent an interesting subgroup as they consist of small molecules with a high reactivity in terms of their sulfur components, which replace oxygen in the atmosphere and in the human body [175,176,177]. Environmental sulfides are often found as large-area sources [178,179,180,181,182]. Additionally, sulfides also produce other sensory effects, including a characteristic olfactory response. Because of the sensory effects related to odors, ATSDR developed an environmental odors website to assist in addressing their impacts on olfaction and limbic systems, as well as on the other health endpoints [183].

Carbon disulfide is a well-studied ototoxicant, targeting cochlear cells, that causes hearing loss and impacts the vestibular system [184,185,186,187]. Carbonyl sulfide studies suggest ototoxic potential, with effects on brainstem regions that are associated with auditory signal transmission [188,189,190]. In contrast, studies of hydrogen sulfide indicate protective effects on hearing, while having other known neurological effects [191,192]. Sulfur dioxide is a common air pollutant highly associated with respiratory impact. While sulfur dioxide impacts mucociliary clearance in the respiratory system, it was not found to in the tympanic cavity of the middle ear [127]. Thus, while general air pollution that includes sulfur dioxide might have an association with hearing loss, especially in patients with Meniere’s disease, this could be due to other air pollutants like carbon monoxide or oxides of nitrogen within the mixture that do have a mechanism of ototoxicity [193,194,195,196,197,198].

#### 4.1.5. Metals

The classes of chemicals investigated as potential ototoxicants (organic solvents, heavy metals, nitriles, organotins, asphyxiants, and pesticides) have diverse structures, suggesting several targets for injury within the auditory system and an array of possible underlying mechanisms. Metals may affect both the peripheral (cochlea) and the central auditory pathways. Lead is the most extensively studied metal in relation to ototoxicity, more than other toxic metals such as cadmium or mercury. Lead is commonly found in occupational and community settings due to its use in lead–acid storage batteries and past contamination from gasoline, plumbing, and paint. However, many scenarios of environmental or occupational exposure to other metals are also common and of concern in terms of ototoxicity [132]. Such sources of exposure include industrial activities, mining operations, contaminated soil and water, air pollution, and consumer products. While lead levels in the air have dropped since it stopped being added to gasoline and paint, childhood lead exposure continue from soils and from older homes with lead paint and plumbing [199].

In the environment, metals can persist for long periods and accumulate in ecosystems. They can enter the food chain through plants or animals, leading to potential human exposure through the consumption of contaminated food or water. Lead and cadmium exposures therefore occur at all ages. Additionally, certain hobbies or cultural practices involving the use of metals (e.g., pottery glazes containing lead) can also contribute to exposure. Cigarette and tobacco products expose people to cadmium and other metals [200,201].

Metals with lower ototoxic potential might offer protection from more toxic metals when they compete for uptake in the human body and distribution to organs. Some forms of zinc, copper, and iron compete with cadmium and lead, all common in the environment. People with low nutrition levels are highly susceptible to lead uptake, in part due to the balance of zinc and other beneficial metals [202]. While zinc and copper provide an uptake benefit, copper can be toxic [203,204]. The ability of heavy metals to compete with each other was demonstrated by using copper sulfate to reduce the dose of platinum reaching the cochlear cells, which was associated with hair cell death and hearing loss [205]. While it is helpful to reduce the risk of platinum-induced ototoxicity, copper’s ototoxicity potential is still uncertain; however, it is expected to be very limited as copper is a neurotoxicant at high doses and the only cases where dizziness was reported was following high acute exposures. It is assumed that copper sulfate overactivates vestibular system inputs resulting in nausea and vomiting, thus protecting mammals from copper toxicity [206,207]. Copper is also toxic to the liver and kidneys, organs with known associations with ototoxicity [208,209,210].

Zinc competes with cadmium, reducing cadmium toxicity [205,211,212]. Metallothionein regulates the metabolism of both zinc and cadmium. When cadmium binds to metallothionein, it has toxic effects on tissues. However, metallothionein also increases the transport of cadmium to the kidneys. Metallothionein can also bind copper, mercury, and selenium [213]. Cadmium toxicity has been observed in rat cochlear hair cells, auditory nerve fibers, and spiral ganglion neurons as well as in zebrafish lateral line cells [214,215]. The mechanisms of cadmium ototoxicity suggest a redox imbalance.

There is inconsistent evidence that higher cadmium exposures result in hearing loss, and clear dose–response relationships are missing. Cadmium, like many of the substances explored in this review, is an illustrative example of a substance with a persisting ototoxicity research gap. More consistent evidence into both humans and animals would improve the understanding of mechanisms linked to cadmium ototoxicity and its dose–response relationship, given its known risk to workers and other persons exposed to cadmium. The *Toxicological Profile for Cadmium* reviewed in this study was published in 2012 and did not reflect the most recent evidence on cadmium ototoxicity.

#### 4.1.6. Noise

Loud noise is the greatest contributor to hearing loss. But moderate noise can also impact hearing and have other ototoxic effects when someone is exposed to an ototoxicant [20,216]. Noise causes mechanical and metabolic damage to the peripheral auditory receptor, the cochlea. This can result in cochlear damage, vestibular damage, and at times damage to the auditory neural pathways. Noise increases blood flow to the cochlear hair cells, permitting any ototoxicants in the blood to target those cells. High levels of noise exposures can cause damage to cochlear outer hair cells, leading to increased thresholds (hearing loss); tinnitus; and damage to nervous system tissues within the central auditory system, leading to hyperacusis [217,218].

### 4.2. Toxicological Research and Tinnitus

Toxicological studies for tinnitus are rare, and any dose–response data for animals are not easily translatable to the human experience. While some cases of tinnitus can be explained by physical malformations, many cases can only be detected by an individual’s senses [219]. Thus, it is most often studied in self-reported surveys. This is in stark contrast to studying hearing loss, which can be measured with audiometric testing or by examining damage to inner ear cells, such as the mechanosensory hair cells within the cochlear organ of Corti [220].

Epidemiologic studies find that tinnitus is associated with many of the same exposure factors as hearing loss, but not all. Age and exposure to noise, toluene, lead, and carbon monoxide were associated with self-reported tinnitus in a cross-sectional study of 4970 workers [134]. Exposure to loud noise, heavy metals, solvents, smoke, and exhaust were associated with a 2–4-fold increase in self-reported tinnitus among randomly selected military personnel from two stations (*n = 1833 and 2342*) [221]. Combined exposure to ototoxicants and noise was associated with more moderate and severe tinnitus compared with noise exposure alone.

While our review did not find many case control studies of single-substance exposure-induced tinnitus, there are several studies that relate individual prescription drugs with tinnitus [222,223,224,225,226,227,228,229,230]. Many of the drugs associated with tinnitus are also associated with hearing loss. Some, like statins, while associated with hearing loss, are also associated with a reduction in tinnitus [231,232,233]. This is possibly due to the risk of hypertension and increased tinnitus [234,235,236]. Also, it could be due to the impact of many ototoxic drugs, centered on the structures and sensory cells of the inner ear [237].

## 5. Limitations

This review is limited to 61 profile reports (of 62 substance groups) and is not inclusive of all the substances that ATSDR has completed profiles for or others that ATSDR has placed on the SPL. This is due to resource limitations, the date of some publications, the recency of some ototoxicity data, and the selected substance categories. Other profiles are mentioned, but not tabulated. While the profiles provide summaries of health outcomes and risks for many human organs and systems close to their publication dates, they are updated based on a prioritization matrix that accounts for the frequency of exposure. Thus, some profiles might not be updated for years. This is particularly important for health issues that have only just recently been studied, such as ototoxicity. Sometimes the needed updates are captured by closely related profiles, as with parathion (2017), which captured some needed ototoxicity data for methyl parathion (2001) as well as other organophosphorus pesticides [161,165,238,239]. We indicated these cases in the right column of the table (Ototoxicity Confirmation and Supporting Data). As new profiles are published, they may include improvements that will follow a pattern described in this summary, with the neurological effects section and vestibular function expanded as described below. Those new substance profiles will replace the older ones listed in Table 1. Further, our assessment of exposure routes identifies the relative frequencies of exposure for each substance or substance group independently of health outcomes. The order displayed in the table therefore does not necessarily represent the main exposure routes for ototoxicity.

## 6. Recent Profile Advances

Historically, auditory health outcomes involving hearing and balance functions have been mentioned in toxicological profiles and other authoritative sources under the neurological health effects section or neurotoxicity category. This may have contributed to an underestimation and/or underrepresentation of a significant public health problem. Charting the evidence of ototoxicity for toxic substances separately might impact their recognition and prompt further research.

Recent profiles have been indexed to increase the efficacy of word searches in addition to adding ototoxicity-specific language. Hearing professionals were engaged during the last three years while charting the ototoxicity of these hazardous substances. To address their concerns regarding quickly identifying ototoxicity study summaries, ATSDR added the term “ototoxicity” to describe any such effects and may add a ***neurosensory*** subsection when there are sufficient study data to support it. The 1,1,1-Trichloroethane Profile includes three animal studies and a cross-sectional occupational study on hearing loss and ocular-related vestibular effects [240,241,242,243]. While a few studies suggest that equilibrium and coordination are impacted from acute exposures, it is likely that these effects are not permanent and they are likely to be linked to neuromotor (intoxication) not neurosensory effects [244,245,246].

The ototoxicity study data reviewed in developing the recent Hexachlorocyclohexane (HCH) Profile were too limited for a neurosensory subsection to be added. However, word searching within the document for “ototoxicity” and “hearing loss” identifies one worker study, suggesting hearing loss associated with exposure to α-HCH, and one developmental study, associating blood β-HCH and cochlear deficits with mixed findings [125,247]. The latter finding was also reflected as a critical data need in the profile.

## 7. Considerations

Currently, the only hearing test required by the Organization for Economic Cooperation and Development (OECD) when a chemical is to enter the market is the qualitative assessment of the startle reflex (115 dB SPL click). This test is not sufficiently sensitive for the detection of ototoxicity (presented as an abstract) [248]. For this reason, existing ototoxicity information is restricted to a limited number of substances.

## 8. Recommendations

The charted substances provide several resources for researchers, health care providers, exposure scientists, and health and safety officers. These substances are linked to the full profiles so that detailed summaries of all health effects can be reviewed. In time, the newer profiles will contain additional health research.

**Researchers**: Scientists involved with ototoxic research can easily identify potential and possible ototoxicants that have critical data gaps. Exposure histories can be cross-referenced with tables to identify potential ototoxic risks, as well as other associated effects. This cross-referencing can facilitate the understating of the underlying mechanisms of toxicity. Should dose–response data be obtained with and without noise, they can be used to establish safe exposure levels.

**Healthcare providers**: Clinicians and audiologists involved in care for patients with hearing loss or other ototoxic effects are likely familiar with the OTO designation, but not familiar with ATSDR’s Toxicological Profiles. The profiles and this publication can now be used as a resource guide to link to substances that might be associated with their patients’ clinical history. The table and associated profiles could provide additional data to help in evaluating exposure histories. ATSDR provides a portal linking several target organ systems with toxic substances that, at sufficient exposure levels, may potentially harm them: Health Effects of Exposure to Substances and Carcinogens|Toxic Substance Portal|ATSDR (cdc.gov) (accessed on 29 August 2024).

Individuals may unknowingly be exposed to ototoxicants not listed as OTOs. For instance, morticians and agricultural workers are likely exposed to formaldehyde; drycleaners and degreasers are likely exposed to tetrachloroethylene. Furthermore, cleaning and beauty salon employees likely have acetone exposure and metalworkers may be exposed to manganese, lead, and chromium. Several hazardous waste sites also report these chemicals and other potential ototoxicants as being among those substances to which members in neighboring communities are frequently exposed. It is possible that the early prevention and mitigation of ototoxic effects might serve to prevent other serious health outcomes.

**Exposure scientists**: Exposure scientists tasked with evaluating exposure data and determining the specific target organs and systems have little data for evaluating hearing loss or any ototoxic effects. Very few studies on any of these substances provide sufficient dose–response data to estimate risk. As a result, exposure scientists typically select another health endpoint to address. With other neurologic effects, like tapping response, being a more frequently assessed health response, those risks are typically used to develop health risk messaging. By using the “other health endpoint approach”, scientists avoid considering how even a small amount of noise, combined with exposure to the substance, might increase the harmful effects.

**Health and safety officers**: Health and safety officers are tasked with protecting workers from exposure to hazardous substances. Many substances that present some evidence of ototoxicity risk are not designated as OTOs. Thus, it is not likely that the material data safety sheet will include ototoxicity information to inform workers of that potential risk. The substance chart introduces the ATSDR’s Toxicological Profiles, identifying those with evidence of ototoxicity and where these data might be found for most substances.

## 9. Challenge to Researchers

Because of the associations between hearing loss and tinnitus with other serious health outcomes and the associations between ototoxic substances and those serious health outcomes, research is needed to explore the causal relationships. While cochlear hair cell damage is permanent, the early detection of hearing damage can result in interventions to delay progression to more serious hearing effects. Yet, auditory testing is limited. Periodic auditory screening efforts could prevent hearing loss in more individuals. If the loss was due to ototoxic exposures, this could help to prevent other serious health outcomes associated with exposure to those substances. Currently, no ATSDR MRL is based on ototoxicity research due to the lack of dose–response data. Audiometric measures could easily be included, along with other neurologic endpoints. It is possible that, in some cases, ototoxicity is a more sensitive endpoint that would result in the calculation of more protective exposure doses.

## Figures and Tables

**Table 1:** Summary of evidence base for developmental, neuro-, renal-, nephro-, and oto- toxic potential of hazardous substances found frequently in the environment, by exposure route, as identified in the ATSDR Toxicological Profiles.

Toxicological Profile (Date)	Hearing Loss	Tinnitus	Vestibular	Exposure Route (Most to Least Frequency)	Hepatotoxicity	Renal and Nephrotoxicity	Neurological	Developmental	Ototoxicity Confirmation and Supporting Data
Fuels and Oils									
BTEX interaction (2004) petroleum products gasoline & coal	Y	NA	P	I, O, D	Y	Y	Y	Y	Hearing loss and dizziness are listed throughout. See individual components in VOCs.
Fuel Oils (1995) [59]	P; for kerosene	NA	P; (Sec 2)	I, O, D	Y	DL/IF	Y	DL/IF	P; many neurological effects including ocular (Sec 2 p26, p80); see JP5.
Jet Fuels JP4 & JP7 (1995) [60]	P; (Sec 2.2)	NA	P; (Sec 1.5; 2.2)	I, D, O	Y	DL/IF	Y	DL/IF	P; many neurological effects including ocular (Sec 2.2, 2.2.1.4); see JP5.
Jet Fuels JP5, JP8, and Jet A Fuels (2017) [61]	Y (Sec 3)	NA	Y	I, D, O	Y	DL/IF	Y	DL/IF	Y; hearing loss and altered balance (Sec 3.2.1.4 p53, p55).
Otto Fuel II & components (1995) [62]	NA	NA	P; (Sec 2)		DL/IF	DL/IF	Y	DL/IF	P; many neurological effects, including ocular (Sec 2.2.1.4, p341; Sec 2.2,3,4 p64); see JP5.
Volatile Organic Compounds (VOCs) & Solvents									
1,1,1-Trichloroethane (2024) [63]	Neg (Sec 2.15)	NA	P (equilib Sec 2.15), not ocular	I, O, D	Y	DL/IF	Y	DL/IF	P; but some studies have neg findings (Sec 2.15).
1,2-Dichloroethane (2022) [64]	NA	NA	NA	I, O, D	Y	Y	Y (ataxia Sec 2.15)	Y	NA
2-Butanone (2020) [65]	NA	NA	NA	I, O, D	DL/IF	DL/IF	Y	DL/IF	P; headache, incoordination (Sec 2.15).
Acetone (2021) [66]	Y (Sec 2.18)	NA	NA	I, D, O	DL/IF	Y	Y	Y	Y; hearing loss with mixtures (Sec 2.18).
Acrolein (2024) [68]	NA	NA	NA	I, O, D	DL/IF	DL/IF	DL/IF	DL/IF	NA
**Benzene (2007)** [67]	Y (Sec 2.2)	NA	Y (Sec 3.2.1.4)	I, D, O	DL/IF	DL/IF	Y	DL/IF	**Y (Sec 2.2; 3.2.1.4 p89; Sec 3.2.2.4 p134) & (ASHA/CCOHS)**
**Chlorobenzene (2020)** [69]	Y (Sec 2.2)	NA	NA	I, O, D	Y	Y	Y	DL/IF	** Y (OSHA) **
Chloroethane (2024) [70]	Y (Sec 1.5)	NA	Y (2.2.1.4) NS	I, D, O	DL/IF	Y	Y	Y	P; due to observed vertigo (Sec 2.2.1.4).
Chloroform (2024) [71]	P (Sec 2.2)	NA	P (dizziness Sec 1, 2.15)	I, O, D	Y	Y	Y	DL/IF	P; based on (Hu & Schwarz, 1987) [124]. Electrophysiological evaluation of chloroform-induced inner ear damage.
Chloromethane (2022) [70]	NA	NA	Y (Sec 2.15) NS	I, O, D	Y	Y	Y	DL/IF	P; based on vertigo disturbance (Sec 2.15).
**Ethylbenzene (2010)** [73]	Y (Sec 3.2.1.4)	NA	Y (Sec 3.2.1.4)	I, O, D	Y	Y	Y	Y	**Y (OSHA) & (Sec 3.2.1.4 p66; Sec 3.2.2.4 p83)**
Formaldehyde (1999) [74]	Y; (Sec 2.11.2)	NA	Y (Sec 1.5) NS	I, D, O	Y	Y	Y	DL/IF	Y balance (Sec 2.2.1.4)
**n-Hexane (2024)** [75]	Y (Sec 2.15).	NA	P (dizziness Sec 2.15)	I, D, O	DL/IF	DL/IF	Y	DL/IF	** Y (OSHA) **
**Styrene (2010)** [76]	Y (Sec 2.3)	NA	Y (Sec 2.2)	I, D, O	DL/IF	DL/IF	Y	DL/IF	** Y (OSHA); hair cells in the organ of Corti (Sec 3.2.1.4 p68) **
Tetrachloroethylene (PERC) (2019) [77]	Y Sec (2.2)	NA	NA	I, O, D	Y	Y	Y	DL/IF	Y (Sec3.2.1.4 p74, p76)
**Toluene (2017)** [78]	Y (Sec 3.2.1.4)	NA	Y (Sec 3.2.1.4)	I, D, O	DL/IF	DL/IF	Y	Y	** Y (OSHA) **
**Trichloroethylene (TCE) (2019)** [79]	Y (Sec 2.2)	NA	NA	I, O, D	Y	Y	Y	Y	** Y (OSHA) **
Vinyl Chloride (2023) [80]	P (Sec 3.2.1.4)	NA	NA	I, O, D	Y	DL/IF	Y	Y	P (Sec 2.15); visual effects reported from acute exposures
**Xylenes (2007)** [81]	Y (Sec 2.2)	NA	Y (Sec 3.5.2)	I, O, D	Y	Y	Y	DL/IF	** Y; only p-xylene isomer (OSHA) **
Pesticides, Herbicides, and Barriers									
1,2-Dibromoethane (2018) [82]	NA	NA	NS; (Sec 2.15)	O, I, D	Y	Y	DL/IF	DL/IF	NA; Confusion & brain lesions (Sec 2.15)
Aldrin & Dieldrin (2022) [83]	Neg (Zhang et al., 2021) [125]	NA	NA	O, I, D	Y	DL/IF	Y	Y	One Epi study found no hearing loss (Zhang et al., 2021) [125]; NA (Sec 2.15 p63)
Atrazine (2003) [84]	NA	NA	NA	I, O, D	Y	Y	DL/IF	Y	NA
Chlordane (2018) [85]	NA	NA	NA	I, O, D	Y	DL/IF	Y	Y	Dizziness (Sec 2.15)
Chlorpyrifos (1997) [86]	NA	NA	P, NS	I, D, O	DL/IF	DL/IF	Y	DL/IF	Blurred vision (Sec 2.2.1.2)
**Cyanide (2006)** [87]	Y; Sec (2.2)	NA	NA	I, D, O	DL/IF	DL/IF	Y	DL/IF	** Y (Sec 3.12.2) Y (NIOSH) **
DDT, DDE, & DDD (2022) [88]	NA	NA	NA	O, I, D	Y	DL/IF	Y	Y	One breast-feeding study found no hearing loss in the children of exposed workers (Ribas-Fito et al., 2003) [126] dizziness (Sec 2.15 p147).
Diazinon (2008) [89]	NA	NA	P, NS	I, D, O	DL/IF	DL/IF	Y	DL/IF	Dizziness and blurred vision (Sec 3.2.1.4).
Disulfoton (2022) [90]	Y Sec (2.15)	NA	NA	I, O, D	DL/IF	DL/IF	Y	Y	Y (Sec 2.15 p73)
Endosulfan (2015) [91]	NA	NA	NA	I, D, O	DL/IF	DL/IF	Y	Y	NA (Sec 2.3)
Endrin (2021) [92]	NA	NA	NA	O, I, D	Y	DL/IF	Y	DL/IF	(Sec 2.15 p57) offers related data.
Glyphosate (2020) [93]	NA	NA	NA	O, I, D	Y	Y	DL/IF	DL/IF	NA
Heptachlor & Heptachlor Epoxide (2007) [94]	NA	NA	NA	O, I, D	Y	DL/IF	DL/IF	Y	Neurological alterations (Sec 2.2); neuromotor effects lack studies (Sec 3.12.2).
Hexachlorocyclohexane (2024) [95]	Y Sec (2.15)	NA	NA	I, D, O	Y	DL/IF	Y	Y	Y for α-HCH (Sec 2.15); P for β-HCH (Sec 2.17)
Malathion (2003) [96]	NA	NA	P, NS	I, D, O	DL/IF	DL/IF	Y	DL/IF	Equilibrium and vision impacted (Sec 3.2.2.4)
Methyl parathion (2001) [97]	* Y		P, NS	I, D, O	DL/IF	DL/IF	Y	Y	Updated hearing loss data are provided in the parathion profile
n-Nitroso-n-propylamine (2023) [98]	NA	NA	NA	I, D, O	DL/IF	DL/IF	DL/IF	DL/IF	NA
Parathion (2017) [99]	Y (Sec 1, 2.2, 3.3.2.4)	NA	P, NS	I, D, O	DL/IF	DL/IF	Y	DL/IF	Good primate dosing study also human studies (Sec 3.2.2.4)
Pentachlorophenol (2022) [100]	NA	NA	NA	I, O, D	Y	DL/IF	DL/IF	Y	Ocular effects in workers, animal studies disagree (Sec 2.12).
Phosphate Ester Flame Retardants (2012) [101]	NA	NA	NA	O, I, D	Y	Y	Y	DL/IF	NA (Sec 3.2.2.6)
Toxaphene (2014) [102]	NA	NA	NA	I, O, D	DL/IF	DL/IF	DL/IF	Y	NA; limited neurological studies reported (Sec 3.2.3.4).
Sulfides									
**Carbon Disulfide (1996)** [103]	Y (Sec 2.2.1.4)	NA	NA	I, D, O	Y	DL/IF	Y	DL/IF	** Y (OSHA) **
Carbonyl sulfide (2016) [104]	P (Sec 2.2.1.4)	NA	P;	I, O	DL/IF	DL/IF	Y	DL/IF	Carbonyl sulfide impacts brain stem.
Hydrogen sulfide (2016) [104]	P; (Sec 2.2.1.4)	NA	P;	I, O	DL/IF	DL/IF	Y	DL/IF	Hydrogen sulfide is possibly protective.
Sulfur dioxide (1998) [105]	Neg (Ohashi et al. 1989) [127]	NA	NA	I, O	DL/IF	DL/IF	DL/IF	DL/IF	No effusion found in middle ear (Ohashi et al. 1989) [127]
Metals									
Aluminum (2008) [106]	Y; Sec (2.0)	NA	NA	I, O, D	DL/IF	DL/IF	Y	DL/IF	Limited due to studies not reporting aluminum content of the basal diet.
Arsenic (2007) [107]	Y; Sec (2.0)	NA	Y; limited (Sec 3.2.2.4) NS	I, O	DL/IF	Y	DL/IF	DL/IF	Suspected; limited evidence (OSHA).
Cadmium (2012) [108]	Y; Sec (2.0)	NA	NA	O, I	DL/IF	Y	DL/IF	DL/IF	Suspected; limited evidence (OSHA); very good Epi and animal studies with mixed results.
Chromium (2012) [109]	* P	NA	Y; Limited; NS	O, I, D	DL/IF	DL/IF	DL/IF	DL/IF	P; auditory damage in rats (EPA, Zhan et al., 2012) [128]
Cobalt (2023) [110]	P; limited	NA	NA	I, O, D	Y	DL/IF	DL/IF	DL/IF	Potential Y, decreased auditory response, and hearing loss reported during cobalt therapy (Sec 5.7).
Copper (2024) [111]	* P	NA	P; NS	O, I, D	Y	Y	Y	DL/IF	Copper sulfate has potentially protective properties, as demonstrated with platinum-induced ototoxicity.
**Lead (2020)** [112]	Y; Sec (2.0)	NA	NA	I, O, D	DL/IF	Y	Y	Y	** Y (HCA/OSHA) **
Manganese (2012) [113]	Y	NA	Y but limited; NS	I, O	DL/IF	DL/IF	Y	Y	Suspected (Ding et al., 2011) [129]
**Mercury (2022)** [114]	Y; Sec (2.0)	NA	Y	O, I	DL/IF	Y	Y	Y	** Y (HCA/OSHA) **
Nickel (2005) [115]	NA	NA	NA	O, I, D	DL/IF	DL/IF	DL/IF	Y	No ototoxicity studies (Castellanos & Fuente, 2016) [130]
Selenium (2003) [116]	* P	NA	Y	O, I	Y	Y	Y	DL/IF	Treats idiopathic sudden sensorineural HL (Kaya et al., 2015) [131], but higher levels are significantly associated with HL (Carlson, 2019) [132].
**Tin and tin compounds** (2005) [117]	Y; Sec (2.2)	NA	NA	O, D, I	Y	Y	Y	DL/IF	** Y, Organotins (OSHA) **
Zinc (2005) [118]	* P	NA	Y but limited; NS	O, I	DL/IF	DL/IF	DL/IF	DL/IF	P; increasing levels of zinc were associated with HL (Carlson, 2019) [132].

**Table 2:** Health outcome associations for 62 substances.

Ototoxic Potential [62]	Neurotoxic Potential	Renal and Nephrotoxic Potential	Hepatotoxic Potential	Developmental Effects
OTO 13	100%	54%	46%	38%
EO 27	89%	48%	44%	37%
SEOP 41	85%	41%	49%	29%

## Data Availability

The data presented in this study are available on request from the corresponding author.
